# Differences in MAP kinase phosphorylation in response to mechanical strain in asthmatic fibroblasts

**DOI:** 10.1186/1465-9921-7-68

**Published:** 2006-04-27

**Authors:** Frédérique Le Bellego, Sophie Plante, Jamila Chakir, Qutayba Hamid, Mara S Ludwig

**Affiliations:** 1 Meakins-Christie Laboratories, McGill University Hospital Center, Montreal, Quebec, Canada; 2 Centre de Recherche, Hôpital Laval – Institut Universitaire de Cardiologie et de Pneumologie, Sainte-Foy, Quebec, Canada

## Abstract

**Background:**

Mechanical strain alters protein expression. It results in phosphorylation of MAP kinases and up-regulation of extracellular matrix proteins. We investigated whether phosphorylation of MAP kinase family members was increased in response to mechanical strain in fibroblasts from asthmatic patients (AF) and normal controls (NF), and whether phosphorylation of these signaling molecules would be different in the two cell populations.

**Methods:**

Fibroblasts were obtained from mild, atopic asthmatics and non-atopic volunteers using endobronchial biopsy. Cells were grown on flexible, collagen I-coated membranes, and subjected to mechanical strain (Flexercell). MAP kinase phosphorylation was measured at baseline, and during one hour of strain. We also examined the effect of strain on proteoglycan production.

**Results:**

At baseline, there was increased phosphorylation of ERK1/2 and p38, and decreased phosphorylation of JNK in AF vs NF. During strain in NF, p38 phosphorylation was increased. Conversely in AF, strain resulted in an increase in JNK phosphorylation, had no effect on phosphorylation of p38, and resulted in a decrease in ERK1/2 phosphorylation. There was a significant increase in versican protein production after 24 h strain in both AF and NF. JNK inhibition reversed the strain-induced increase in versican in NF, but had no effect in AF.

**Conclusion:**

These results show that there are phenotypic differences in MAP kinase phosphorylation in AF vs NF, and that different signaling pathways are involved in transducing mechanical stimuli in these two populations of cells.

## Background

Mechanotransduction involves the ability of the cell to respond to mechanical strain with a biological message and alteration of protein production. Studies of lung cells *in vitro *have identified some of the intracellular signaling pathways that mediate this effect, which include members of the mitogen-activated protein (MAP) kinase signaling family. Phosphorylation of MAP kinases results in downstream phosphorylation of other signaling molecules, and ultimately, activation of transcription factors [1]. Cyclic stretch activates extracellular signal-regulated kinase (ERK) 1/2 in different types of pulmonary cells, including alveolar and bronchial epithelial cells [2-4] Mechanical strain also enhances p38 activation in bronchial epithelial cells and in parenchymal lung strips [3,5]. Finally, phosphorylation of c-Jun NH_2_-terminal kinase (JNK) is increased in response to mechanical strain in both bronchial epithelial cells and in type II-like alveolar epithelial cells [3,6]. Mechanical strain affects the production of extracellular matrix (ECM) components, upregulating type I collagen in pulmonary fibroblasts, type III and IV collagen in co-cultures of bronchial epithelial cells and lung fibroblasts, and the proteoglycans (PGs), versican, biglycan and perlecan, in human arterial smooth muscle cells [7-9].

The asthmatic airway wall is subject to increased mechanical strain or stress, due to bronchoconstriction of the airways and the heterogeneous distribution of ventilation [10]. Asthmatic airways are characterized by remodeling of the airway wall, with an increased deposition of ECM components including collagen, elastin and PGs [11-13] Mechanical strain could, potentially, be an important stimulus for this airway wall remodeling. Therefore, understanding the mechanisms by which matrix is upregulated in response to mechanical strain in asthmatic airway cells, should give us new insight into asthma pathophysiology. We have recently shown that versican and decorin mRNA is increased in response to mechanical strain in fibroblasts from asthmatic subjects, in comparison to cells from normal controls [14]. Some data is also available in an animal model of asthma, the allergen sensitized mouse. Kumar et al [5] have shown that ERK 1/2 is preferentially upregulated in parenchymal lung strips from sensitized, challenged mice subjected to mechanical stretch, as compared to strips from non-sensitized control mice. There is no data, however, available in human asthmatics.

To investigate these questions in human disease, we obtained fibroblasts from asthmatic patients and normal volunteers using endobronchial biopsy. We studied fibroblasts, as they are the major cell cell type putatively responsible for the airway wall remodeling characteristic of asthma [15]. We questioned whether MAP kinase phosphorylation in response to mechanical strain would be similar in fibroblasts from asthmatic patients (AF) as compared to fibroblasts from normal controls (NF), and whether this mechanical signal would result in upregulation of PG protein.

## Methods

### Materials

The following reagents were obtained from Sigma (Oakville, Ont., Canada): EDTA, EGTA, Triton X-100, sodium pyrophosphate, β-glycerophosphate, sodium orthovanadate (Na_3_VO_4_), sodium fluoride (NaF), protease inhibitor cocktail, phenylmethylsulfonyl fluoride (PMSF), Bio-Rad reagent, Tween_20_, Guanidium-HCl, 6-aminohexanoic acid, benzamidine hydrochloride, N-ethylmaleimide, JNK inhibitor (SP 600125) and antibody against actin. Dimethylsulfoxide (DMSO) was obtained from Fisher Scientific. Fetal calf serum (FCS) came from HyClone (Logan, UT, USA). Dubelcco's modified Eagle's medium (DMEM), penicillin G, streptomycin, amphotericin B, trypsin came from Gibco-BRL-Invitrogen (Burlington, Ont., Canada). Nitrocellulose and polyvinylidene difluoride (PVDF) membranes, streptavidin-biotinylated horseradish peroxidase (streptavidin-HRP), chemiluminescence reagent (ECL+ and ECL assay) were obtained from Amersham Biosciences Corp. (Piscataway, NJ, USA). The antibodies: rabbit anti-phosphorylated ERK1/ERK2, anti-phosphorylated p38, anti-phosphorylated JNK, anti-total ERK1/ERK2 and anti-total p38 came from Cell Signaling Technology (Beverly, MA, USA), mouse anti-human fibroblast antigen Ab-1 antibody from Calbiochem (San Diego, CA, USA), biotin-labeled swine anti-rabbit secondary antibody and biotinylated rabbit anti-mouse secondary antibody from DAKO (Mississauga, ON, Canada), monoclonal mouse anti-versican antibody (12C5) from Developmental Studies Hybridoma Bank (Iowa City, IA, USA), rabbit anti-decorin from Dr Larry Fisher [16,17]., and rabbit anti-lumican, a gift from Dr Peter Roughley (Shriner's Hospital, McGill University). BioFlex silastic-bottom culture plates were from Flexcell International Corp. (McKeesport, PA, USA).

### Bronchial fibroblast cell lines

Primary fibroblasts were isolated from bronchial biopsies of 8 asthmatic patients and 8 healthy volunteers (Table 1). All patients gave written informed consent, as approved by the Laval Hospital Ethics Committee. Asthmatic patients had mild disease, as characterized by the use of β agonist only. None had ever used inhaled or systemic corticosteroids. All asthmatic patients were non-smokers, and atopic, confirmed with a positive skin reaction to at least one common allergen. Patients had PC_20 _methacholine ranging from 0–4.21 mg/ml and FEV_1 _within the normal range. All normal subjects were non-atopic, non-smokers and had PC_20 _methacholine greater than 16 mg/ml. Additional details on selection and evaluation of subjects, bronchoscopy and bronchial biopsy procedures, biopsy processing, identification and characterization of bronchial fibroblasts have been described in previous publications [18-20] Isolated fibroblasts were characterized by immunofluorescence and flow cytometry using a mouse anti-vimentin antibody, and a mouse anti-human fibroblast antigen Ab-1 antibody that shows no cross-reactivity with epithelial cells, endothelial cells, smooth muscle cells, or other cell types. This identification confirmed the purity of bronchial fibroblast cell culture [21]. Cells were used at fifth or sixth passage.

**Table 1:** Subject Characteristics

Group	Sex	Age	PC_20_	FEV_1_(%)	Atopy	Medication
Asthmatic	M	24	2.33	4.03 (98)	Yes	β-agonist only
Asthmatic	F	20	4.21	3.51 (105)	Yes	β-agonist only
Asthmatic	M	20	2	4.22 (98)	Yes	β-agonist only
Asthmatic	F	18	0.56	3.09 (36)	Yes	β-agonist only
Asthmatic	M	18	0.66	4.29 (94)	Yes	β-agonist only
Asthmatic	F	22	0.14	3.55 (93)	Yes	β-agonist only
Asthmatic	M	26	saline	3.77 (82)	Yes	β-agonist only
Asthmatic	M	26	3.11	4.00 (76.7)	Yes	β-agonist only
Mean		21.8 ± 3.3	1.9 ± 1.5	3.8 ± 0.4		

Normal	M	32	> 128	3.76 (94)	No	No
Normal	F	38	> 128	3.27 (n/a)	No	No
Normal	M	23	> 128	n/a	No	No
Normal	M	50	> 256	4 (80)	No	No
Normal	M	32	25.5	4.01 (122)	No	No
Normal	F	26	96	2.93 (95)	No	No
Normal	M	29	17.7	3.82 (89)	No	No
Normal	M	23	> 128	5.06 (103)	No	No
**Mean**		31.6 ± 9.0		3.8 ± 0.7		

PC_20_: provocative concentration of methacholine to cause a fall in FEV_1 _of 20%; FEV_1_: forced expiratory volume in 1 second; % predicted is shown in parentheses; M, male; F, female; n/a, not available

### Cell culture

Fibroblasts were cultured in DMEM supplemented with 10% heat-inactivated FCS, 100 U/ml penicillin G, 100 μg/ml streptomycin and 250 ng/ml amphotericin B at 37°C in the presence of 5% CO_2_. Sub-culturing was carried out using trypsin (0.25%).

### Mechanical stimulation of cultured fibroblasts

Cells were seeded on type I collagen-coated 6-well BioFlex silastic-bottom culture plates at a concentration of 1.5 × 10^5 ^cells/well. Cells were grown in the BioFlex plates until ~ 90% confluence. Cells were serum-starved by replacing the medium with DMEM without FCS, with antibiotics and antimycotics for 24 h. Plates were then transferred to the baseplate of the cell stretching device (FX-3000 Flexercell strain unit, Flexcell International, McKeesport, PA) and placed in a 37°C, 5% CO_2 _incubator. Application of a negative pressure caused a downward deformation of the flexible silastic membrane to which the cells were attached. A biaxal strain of 30% amplitude at a frequency of 1 Hz was applied for either 1 or 24 hr. We chose this regimen of strain based on previous experiments conducted in a fibroblast cell line [22,23] and in fibroblasts isolated from normal and asthmatic patients [14]. At this amplitude of strain, increases in versican protein and mRNA were maximal. As we were interested in the signaling pathways contributing to the proteoglycan signal, we thought it most appropriate to use this regimen. Control cells were cultured in BioFlex plates but not submitted to cell stretch (static condition). Cell layer and/or supernatants were harvested at various time points, and processed. Cell viability was assessed by Trypan blue exclusion.

### MAP kinase expression

MAP kinase expression was assessed at baseline and then after stretching for 10, 20, 30, and 60 min. Cells were washed twice in ice-cold PBS and scraped in lysis buffer containing 20 mM Tris, pH 7.5, 150 mM NaCl, 1 mM EDTA, 1 mM EGTA, 1% Triton X-100, anti-phosphatases: 2.5 mM sodium pyrophosphate, 1 mM ^β ^-glycerophosphate, 1 mM Na_3_VO_4_, 1 mM NaF, and protease inhibitor cocktail. Protein content was determined by the Bradford technique, using a Bio-Rad protein assay system. Protein (10 μg) was resolved in a 12% polyacrylamide gel and transferred onto PVDF membranes. Membranes were immunoblotted overnight, at 4°C, with 1/1000 dilution of rabbit anti-phospho-MAP kinases antibodies (anti-phosphorylated ERK1/2, anti-phosphorylated p38 and anti-phosphorylated JNK). After being washed with Tris-buffered saline with Tween_20 _(TBST), membranes were incubated with a 1/1000 dilution of biotin-labeled swine anti-rabbit secondary antibody for 1 h at room temperature, followed by 1 h incubation with streptavidin-HRP (1/5000) in TBST. Detection was performed using enhanced chemiluminescence (ECL+ assay). To ensure equal loading and protein transfer, membranes were submitted to a stripping protocol by incubation for 30 min at 60°C in a buffer containing 62.5 mM pH 6.8 Tris-HCL, SDS 2%, and 100 mM β-mercaptoethanol, and reprobed using anti-"total" MAPK antibodies (anti ERK1/2, anti-p38 or anti-actin). Second steps and detection were as described above. The densitometric values for phosphorylated forms of MAP kinases were normalized according to the values for total MAP kinases or actin. Separate sets of cells were used for measurement of MAP kinase expression at baseline, and each time point after stretch.

### PG extraction and expression

Cells were submitted to cell stretching for 24 h. After cyclic stretch, medium was aspirated and stored at -20°C. The cell layer was rinsed three times with PBS. PGs were extracted with ice-cold 4 M Guanidium-HCl-50 mM sodium acetate (pH 5.8)-1% Triton X-100 containing the following proteases inhibitors: 100 mM 6-aminohexanoic acid, 10 mM EDTA, 5 mM benzamidine hydrochloride, 10 mM N-ethylmaleimide, 0.1 mM PMSF at 4°C overnight. The PGs extracts were then centrifuged at 15000 rpm for 30 min; the supernatants were dialyzed exhaustively against 50 mM Tris-HCl (pH 8.0) containing proteases inhibitors and distilled water, and concentrated; protein content was measured (Bio-Rad protein assay). For measurement of versican, PG was extracted from the medium; for measurement of lumican and decorin, PGs was extracted from the cell layer. Electrophoretic separation of versican was performed in 5% SDS-Page, and small PGs (lumican and decorin) in 10% SDS-Page. After electrophoresis, separated PGs were transferred to nitrocellulose membranes at 30 volts overnight at 4°C. After blocking, membranes were probed with mouse monoclonal anti-versican antibody, rabbit polyclonal anti-decorin or rabbit polyclonal anti-lumican (1:1,000) for 1 h at room temperature. After washing with TBST, membranes were incubated with a biotinylated rabbit anti-mouse or swine anti-rabbit secondary antibody (1:1,000) for 1 h at room temperature, washed again with TBST, and then incubated in streptavidin-HRP (1:1,000) for 1 h at room temperature. After washing of membranes, antibody binding was visualized through ECL detection.

### JNK inhibition

In a subset of experiments, the JNK inhibitor, SP600125 (20 μM), dissolved in DMSO, or DMSO alone, was added thirty minutes before stretching for 24 hr with the Flexercell device. A third set of cells subject to stretch received neither SP600125 nor DMSO. Experiments were carried out in both AF and NF, with and without strain. To check for successful JNK inhibition, cells pre-treated with either SP600125 or DMSO for 30 min, were stimulated with sorbitol (which stimulates JNK phosphorylation). Pre-treatment with SP600125, but not DMSO, abrolished the sorbitol-induced increase in JNK phosphorylation.

### Quantification of immunoblots

Densitometric analysis was performed using image analyzer software, the FluorChem^tm ^FC 800 system (Alpha Innotech, San Leandro, CA, USA), which measures the sum of all the pixel values after background correction.

### Statistical analysis

The data were analyzed using GraphPad software. Data are reported as mean ± standard error. ANOVA with Dunnett's multiple comparison tests was used to analyze differences within groups at different time points. T test was used to compare data between groups.

## Results

### At baseline, MAP kinase activation is increased in AF vs NF

**Figure 1:**
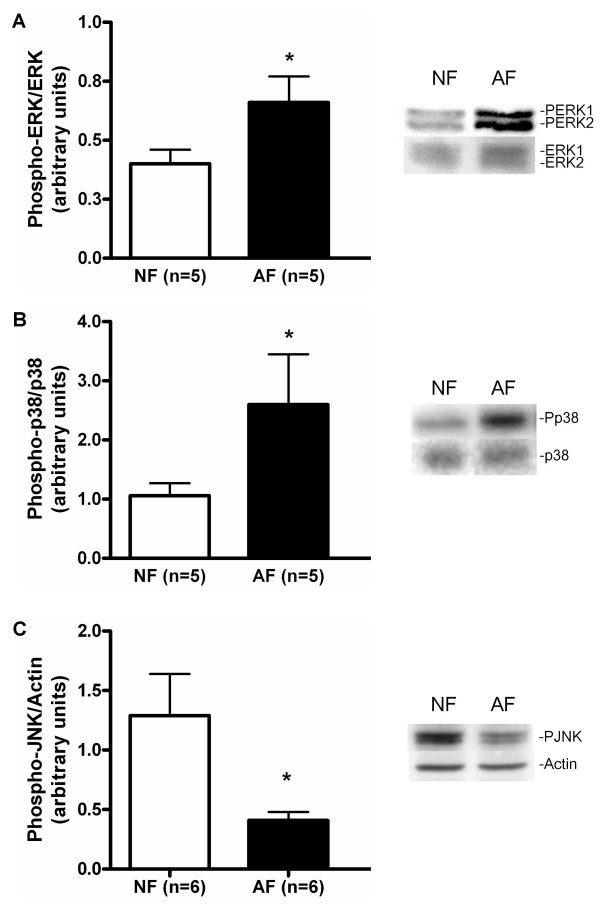
Phosphorylation of MAP kinase family proteins at baseline. Phosphorylation of the MAP kinases was detected by immunoblot on cell lysates using antibodies specific to the phosphorylated forms of the kinases (right panels). Equal loading of cell lysates was determined by immunoblotting with antibodies raised against unphosphorylated forms or actin. Left panels show the quantification of (A) ERK1/2, (B) p38 and (C) JNK. The values for phosphorylated forms of each MAP kinase were normalized according to the values for the respective, total MAP kinase or actin (JNK). The data represent mean ± SE. AF, fibroblasts from asthmatic patients; NF, fibroblast from normal controls. *: p < 0.05

At baseline, the phosphorylation of ERK1/2 was increased 1.65 fold in AF in comparison to NF (Figure 1A, p < 0.05). The phosphorylation of p38 was 2.45 fold greater in AF than in NF (Figure 1B, p < 0.05). There was decreased phosphorylation of JNK (3.14 fold) in AF in comparison to NF (Figure 1C, p < 0.05).

### Mechanical strain resulted in differential phosphorylation of MAP kinases in AF vs NF

**Figure 2:**
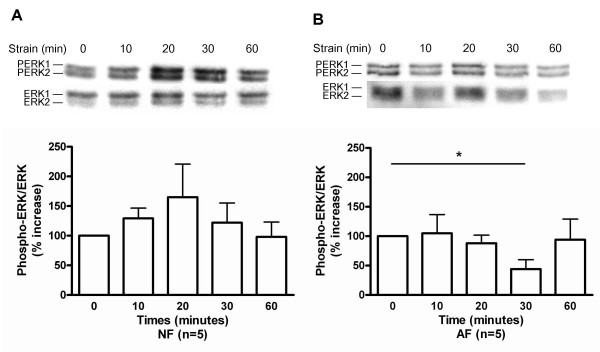
Effect of strain on ERK1/2 phosphorylation in (A) normal (NF); and (B) asthmatic fibroblasts (AF). Phosphorylation of ERK1/2 was detected by immunoblot on cell lysates using antibodies specific to the phosphorylated forms of ERK1/2 (upper panels). Equal loading of cell lysates was determined by immunoblotting with antibodies raised against total ERK1/2. Lower panels show the quantification of ERK1/2 bands. The values for phosphorylated forms of ERK 1/2 were normalized according to the values for total ERK1/2. The data represent mean ± SE. *: p < 0.05

**Figure 3:**
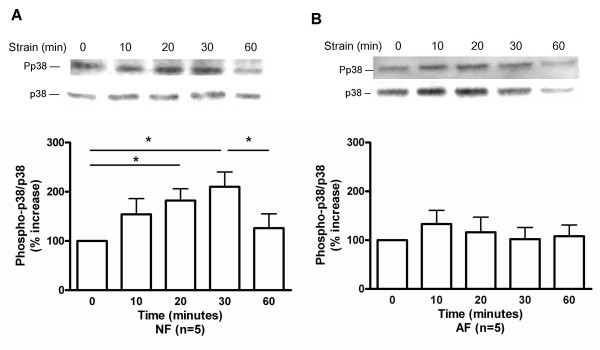
Effect of strain on p38 phosphorylation in (A) normal (NF) and (B) asthmatic fibroblasts (AF). Phosphorylation of p38 was detected by immunoblot on cell lysates using antibodies specific to the phosphorylated form of p38 (upper panels). Equal loading of cell lysates was determined by immunoblotting with antibodies raised against total p38. Lower panels show the quantification of p38 bands. The values for phosphorylated p38 were normalized according to the values for total p38. The data represent mean ± SE. *: p < 0.05.

**Figure 4:**
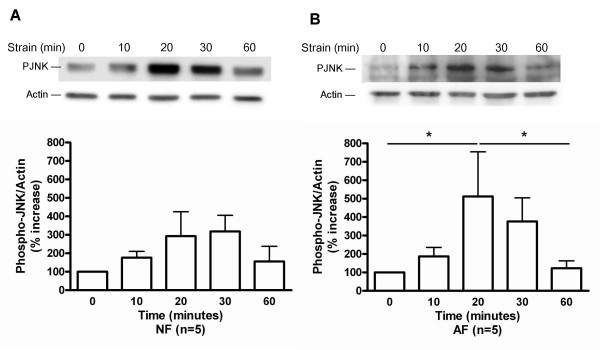
Effect of strain on JNK phosphorylation in (A) normal (NF) and (B) asthmatic fibroblasts (AF). Phosphorylation of the JNK was detected by immunoblot on cell lysates using antibodies specific to the phosphorylated form of JNK (upper panels). Equal loading of cell lysates was determined by immunoblotting with antibodies raised against actin. Lower panels show the quantification of PJNK and actin bands. The values for phosphorylated JNK were normalized according to the values for actin. The data represent mean ± SE. *: p < 0.05

In NF, there was a trend to increased ERK1/2 phosphorylation with a maximum occurring at 20 min of strain (Figure 2A). Conversely, in AF, mechanical strain resulted in a significant decrease in the ERK1/2 phosphorylation at 30 min with a return to basal levels after 1 h strain (Figure 2B). Strain significantly increased p38 phosphorylation in NF (maximum at 30 min) (Figure 3A) (p < 0.05), but had no effect on p38 phosphorylation in AF (Figure 3B). In NF, there was a trend towards increased JNK phosphorylation with a maximum at 30 min of strain (Figure 4A). The JNK phosphorylation in AF was significantly increased, with a maximum occurring at 20 min of strain, and a return to basal levels after 1 h strain (Figure 4B) (p < 0.05).

### Mechanical strain increased versican protein production in both AF and NF

**Figure 5:**
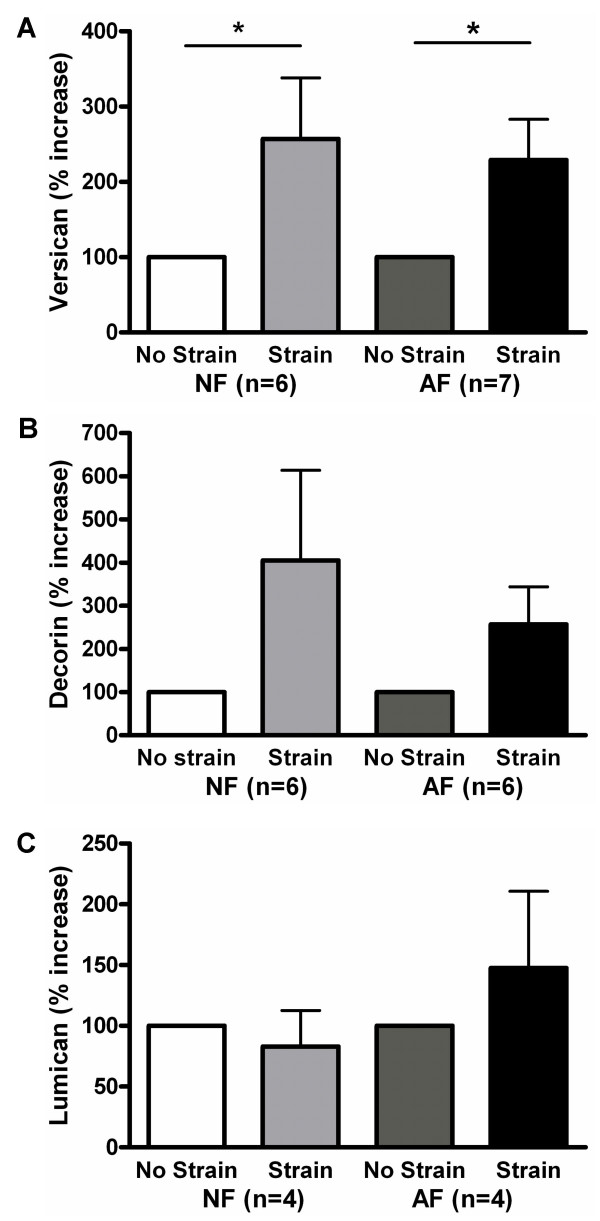
Effect of strain on proteoglycan production in normal (NF) and asthmatic fibroblasts (AF). (A) versican, (B) decorin and (C) lumican production was detected by immunoblot in cell layer or in medium extracts. The data represent mean ± SE. *: p < 0.05

Versican secretion in the medium was significantly increased with strain in both NF and AF (Figure 5A). Decorin production was measured in the cell layer; there was a trend towards increased production of decorin with strain in both AF and NF, but the increase did not reach significant levels (Figure 5B). There was no effect of strain on lumican production (Figure 5C).

### Inhibition of JNK abrogated increased versican production in NF only

**Figure 6:**
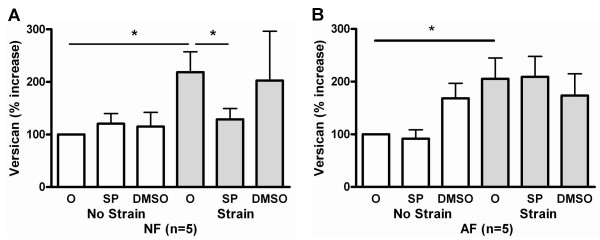
Effect of JNK inhibition on strain-induced versican production in (A) normal (NF) and (B) asthmatic fibroblasts (AF). Versican was increased in response to mechanical strain in both sets of cells. However, JNK inhibition with SP600125 reversed the increase in NF only. Incubation with the vehicle, DMSO, had no effect. The data represent mean ± SE. *: p < 0.05

Addition of JNK inhibitor (SP600125, 20 μM) or DMSO alone, in the culture medium had no effect on versican or decorin production in non-strained cells (both NF and AF). With strain, the previously demonstrated increase in versican production was again demonstrated (p < 0.05). The addition of JNK inhibitor reversed the strain-induced increase in versican production in NF (Figure 6A), but not in AF (Figure 6B). JNK inhibition had no effect on decorin production (data not shown).

## Discussion

The present study reports the first evidence that phosphorylation of MAP kinase signaling proteins is different in fibroblasts from asthmatic patients, compared to fibroblasts from normal volunteers. We also show that JNK phosphorylation in response to excessive mechanical strain, is increased in asthmatic vs normal fibroblasts. Finally, we confirm that mechanical strain increases versican production in both asthmatic and normal fibroblasts, and that the signaling pathways involved in this response are different in these two cell populations.

The increased phosphorylation of ERK1/2, and p38 at baseline in AF supports the hypothesis that MAP kinase signaling represents an "important point of convergence" for the various signaling pathways that are involved in the inflammatory process underlying asthma [24]. *In *vitro studies show that Th2 cytokines, such as interleukin (IL) 4 and 13, known to be upregulated in asthma, activate MAP kinase family members in different types of lung cells. Furthermore, the use of MAP kinase inhibitors reduces or inhibits release of IL-8, and eotaxin, two cytokines implicated in asthma pathophysiology [25,26] ERK 1/2 and p38 have been shown to be involved in eotaxin-induced IL-8 and GM-CSF production by bronchial epithelial cells [27]. JNK has been implicated in the release of GM-CSF, RANTES and IL-8 from bronchial epithelial cells [28]. Laliberté et al [20] have shown that in asthmatic airway fibroblasts there is increased expression of cell surface integrin receptors, as compared to airway fibroblasts isolated from normal volunteers. As integrins activate MAP kinases, the increased number of integrin receptors could potentially contribute to the increased phosphorylation of ERK and p38 observed in AF. Another possibility is that chronic treatment with β-agonists may contribute to increased MAP kinase phosphorylation in AF. A recent paper by Shnackenberg et al [29] reports that isoproterenol increased pERK 1/2 levels in airway epithelial cells. The effect was transient and phosphorylation levels returned to baseline within 30 minutes; hence, we do not believe this mechanism can explain our data.

Studies in animal models of asthma also support the hypothesis that MAP kinases are involved in asthmatic airway inflammation. Duan and co-authors [30] showed that administration of the ERK1/2 inhibitor, U0126, to ovalbumin (OVA) challenged mice, reversed the OVA-induced increases in total cells and eosinophils. The same group also studied bronchial rings from OVA sensitized guinea pigs. Bronchial contractions and release of histamine and peptidyl-leukotrienes in response to OVA challenge were similarly suppressed by U0126 pre-incubation [31]. Eynott and colleagues recently showed that administration of the JNK inhibitor, SP600125, to allergen challenged, sensitized rats decreased macrophage, lymphocyte, eosinophil and neutrophil numbers in BAL [32].

Mechanical stimulation activates MAP kinase signaling proteins. In this study, we show that mechanical strain increased the phosphorylation of p38 in a time-dependent manner in NF. This result confirms data reported in different types of cells, submitted to different types of stress. In lung alveolar epithelial cells, Correa-Meyer et al [2] demonstrated that cyclic stretch induced rapid increases in ERK1/2 phosphorylation. Apical-to-basal transcellular pressure applied to normal bronchial epithelial cells increased ERK1/2 phosphorylation, but not p38 or JNK [4]. The ECM environment can influence mechanical strain-induced phosphorylation of MAP kinase family proteins. MacKenna and collaborators [33] showed integrin- and matrix dependent phosphorylation of ERK1/2, p38 and JNK in rat cardiac fibroblasts. Katsumi and colleagues showed that mechanical stretch stimulation of JNK was dependent on integrin binding to ECM proteins [34]. Phosphorylation of the different proteins of the MAP kinase family in normal cells, is not yet fully characterized, but depends upon the type of cells, the ECM environment, and the precise strain applied.

In contrast to the data in normal airway fibroblasts, in AF, mechanical strain had no effect on the activation of p38, and resulted in decreased phosphorylation of ERK1/2. It is possible that ERK1/2 and p38 were already maximally activated in AF at baseline; hence they could not be further activated by the additional stimulus of mechanical strain. Indeed, the maximal absolute values of phospho-p38/p38 and phospho-ERK1/2/ERK1/2 in NF subjected to strain were roughly equivalent to the elevated values of these molecules at baseline in AF. These data are consistent with the idea that a balance between MAP kinases and MAPK phosphatases, enzymes involved in turning off kinase signaling, modulates the level of phosphorylation in these cells. Along these lines, physical forces have been shown to increase MAP kinase phosphatase expression in vascular smooth muscle cells [35]. Another possibility is that strain may have differentially activated phosphatases, in AF. Our data differ from the data recently reported by Kumar et al [5] in a mouse model of asthma. Stretch of whole-lung parenchyma excised from aspergillus/OVA-sensitized and challenged mice, resulted in increases in ERK1/2 phosphorylation. The differences between these data and that of the current study may relate to differences between the mouse model and human asthma, the response of other cell types included in the excised parenchymal strip preparation, the type of strain applied, etc.

Increases in JNK phosphorylation in response to mechanical strain were significant in AF, but not in NF (although a trend to increased JNK phosphorylation was observed). However, whereas JNK inhibition abrogated the mechanical strain-induced increase in versican protein in NF, no effect was observed in AF. We focused on the JNK pathway as, in the asthmatic cells, this was the only MAP kinase which showed increased phosphorylation with application of mechanical strain. As potential differences in phenotype of asthmatic cells was the prime focus of the experiment, we concentrated our interest on this particular pathway. These data represent another example of the differing phenotype of the asthmatic and control cell populations. They may also reflect a greater difficulty in "turning off" an enhanced matrix response to mechanical strain in asthmatic fibroblasts.

PGs are a heterogeneous family of ECM molecules that consist of a core protein to which one or more glycosaminoglycans are covalently attached. They subserve a number of important biologic functions [36]. Because of the hydrophilic structure of PGs, they have the capacity to attract water into the extracellular matrix, thereby altering tissue turgor and the viscoelastic properties of the matrix. This is especially true for the large, hydrophilic PG, versican. In *in vitro *studies, we have shown that specific degradation of PG alters lung tissue viscoelasticity [37]. PGs interact with various cytokines and growth factors and affect cell migration and proliferation. They also play a key role in collagen fibrillogenesis [36]. PGs have been implicated in the airway wall remodeling characteristic of asthma. In postmortem lung tissue from patients dying with fatal asthma, hyaluronan, versican, biglycan and decorin were prominently localized in the airway wall [38,39]. Lumican, biglycan and versican were increased in the subepithelial layer of bronchial biopsies of mild asthmatics patients, in comparison to normal control subjects [40]. Fibroblasts from asthmatic subjects produce more hyaluronan, perlecan, versican and biglycan, than cells from normal subjects [41].

Mechanical strain increased versican protein in both AF and NF. Previous studies have shown that mechanical stimuli are involved in modulating ECM production [42,43] Studies from our laboratory showed an increase in versican mRNA in response to mechanical strain in both NF and AF (the relative increase in signal was greater in AF) and an increase in decorin mRNA in response to mechanical strain in AF [14]. In the current study, the pattern of increase in versican and decorin protein is consistent with the mRNA data, although some degree of post-transcriptional regulation seems likely. The ability of the cells to alter matrix in response to mechanical strain may represent an important regulatory mechanism – by changing the matrix surrounding the cell, the impact of the mechanical signal could be modulated, both in terms of the magnitude of strain to which the cell is exposed, and the specific signaling pathways stimulated [33,34].

## Conclusion

We have shown that MAP kinase phosphorylation is different in AF as compared to NF, and that MAP kinase responses to mechanical strain vary in the two cell populations. Versican protein was increased in response to mechanical strain in both AF and NF; this increase was abrogated by JNK inhibition in NF, only. These data suggest that different signaling pathways may be involved in the response to mechanical strain, in asthmatic disease.

## Abbreviations

AF: fibroblasts from asthmatic patients

ECM: extracellular matrix

ERK1/2: extracellular signal-regulated kinase 1/2

GM-CSF: granulocyte-macrophage colony-stimulating factor

IL: interleukin

JNK: c-Jun NH_2_-terminal kinase

MAP: mitogen-activated protein

NF: fibroblasts from normal controls

OVA: ovalbumin

PG: proteoglycan

RANTES: regulated-uponactivation normal T-cell expressed and secreted

## Competing interests

The author(s) declare that they have no competing interests.

## Authors' contributions

FLB conceived of the study, participated in the design of the study, carried out all the experiments and wrote the manuscript. SP established fibroblast cell cultures. JC participated in the design of the study and helped to draft the manuscript. QH participated in the design of the study and helped to draft the manuscript. MSL conceived of the study, participated in its design and coordination, and wrote the manuscript. All authors read and approved the final manuscript.

## References

[B1] Johnson GL, Lapadat R (2002). Mitogen-activated protein kinase pathways mediated by ERK, JNK, and p38 protein kinases. Science.

[B2] Correa-Meyer E, Pesce L, Guerrero C, Sznajder JI (2002). Cyclic stretch activates ERK1/2 via G proteins and EGFR in alveolar epithelial cells. Am J Physiol Lung Cell Mol Physiol.

[B3] Oudin S, Pugin J (2002). Role of MAP kinase activation in interleukin-8 production by human BEAS-2B bronchial epithelial cells submitted to cyclic stretch. Am J Respir Cell Mol Biol.

[B4] Tschumperlin DJ, Shively JD, Swartz MA, Silverman ES, Haley KJ, Raab G, Drazen JM (2002). Bronchial epithelial compression regulates MAP kinase signaling and HB-EGF-like growth factor expression. Am J Physiol Lung Cell Mol Physiol.

[B5] Kumar A, Lnu S, Malya R, Barron D, Moore J, Corry DB, Boriek AM (2003). Mechanical stretch activates nuclear factor-kappaB, activator protein-1, and mitogen-activated protein kinases in lung parenchyma: implications in asthma. FASEB J.

[B6] Li LF, Ouyang B, Choukroun G, Matyal R, Mascarenhas M, Jafari B, Bonventre JV, Force T, Quinn DA (2003). Stretch-induced IL-8 depends on c-Jun NH2-terminal and nuclear factor-kappaB-inducing kinases. Am J Physiol Lung Cell Mol Physiol.

[B7] Breen EC (2000). Mechanical strain increases type I collagen expression in pulmonary fibroblasts in vitro. J Appl Physiol.

[B8] Swartz MA, Tschumperlin DJ, Kamm RD, Drazen JM (2001). Mechanical stress is communicated between different cell types to elicit matrix remodeling. Proc Natl Acad Sci U S A.

[B9] Lee RT, Yamamoto C, Feng Y, Potter-Perigo S, Briggs WH, Landschulz KT, Turi TG, Thompson JF, Libby P, Wight TN (2001). Mechanical strain induces specific changes in the synthesis and organization of proteoglycans by vascular smooth muscle cells. J Biol Chem.

[B10] Tschumperlin DJ, Drazen JM (2001). Mechanical stimuli to airway remodeling. Am J Respir Crit Care Med.

[B11] Chu HW, Halliday JL, Martin RJ, Leung DY, Szefler SJ, Wenzel SE (1998). Collagen deposition in large airways may not differentiate severe asthma from milder forms of the disease. Am J Respir Crit Care Med.

[B12] Laitinen A, Altraja A, Kampe M, Linden M, Virtanen I, Laitinen LA (1997). Tenascin is increased in airway basement membrane of asthmatics and decreased by an inhaled steroid. Am J Respir Crit Care Med.

[B13] Roche WR, Beasley R, Williams JH, Holgate ST (1989). Subepithelial fibrosis in the bronchi of asthmatics. Lancet.

[B14] Ludwig MS, Ftouhi-Paquin N, Huang W, Page N, Chakir J, Hamid Q (2004). Mechanical strain enhances proteoglycan message in fibroblasts from asthmatic subjects. Clin Exp Allergy.

[B15] Gizycki MJ, Adelroth E, Rogers AV, O'Byrne PM, Jeffery PK (1997). Myofibroblast involvement in the allergen-induced late response in mild atopic asthma. Am J Respir Cell Mol Biol.

[B16] Bianco P, Fisher LW, Young MF, Termine JD, Robey PG (1990). Expression and localization of the two small proteoglycans biglycan and decorin in developing human skeletal and non-skeletal tissues. J Histochem Cytochem.

[B17] Fisher LW, Termine JD, Young MF (1989). Deduced protein sequence of bone small proteoglycan I (biglycan) shows homology with proteoglycan II (decorin) and several nonconnective tissue proteins in a variety of species. J Biol Chem.

[B18] Dube J, Chakir J, Laviolette M, Saint MS, Boutet M, Desrochers C, Auger F, Boulet LP (1998). In vitro procollagen synthesis and proliferative phenotype of bronchial fibroblasts from normal and asthmatic subjects. Lab Invest.

[B19] Goulet F, Boulet LP, Chakir J, Tremblay N, Dube J, Laviolette M, Boutet M, Xu W, Germain L, Auger FA (1996). Morphologic and functional properties of bronchial cells isolated from normal and asthmatic subjects. Am J Respir Cell Mol Biol.

[B20] Laliberte R, Rouabhia M, Bosse M, Chakir J (2001). Decreased capacity of asthmatic bronchial fibroblasts to degrade collagen. Matrix Biol.

[B21] Chakir J, Dube J, Laviolette M, Goulet F, Germain L, Auger F, Boulet LP, Chund k, Adcock IT (1999). Asthma: Mechanisms and protocols. Methods in molecular medecine.

[B22] Al Jamal R, Grover J, Roughley P, Ludwig MS (2001). Alterations in proteoglycan mRNA in human fibroblasts in response to mechanical strain. [abstract]. Am J Respir Crit Care Med.

[B23] Ludwig MS, Garg HG, Roughley P, Hales CA (2003). Proteoglycans, lung physiology and mechanical strain. Proteoglycans in lung disease.

[B24] Pelaia G, Cuda G, Vatrella A, Gallelli L, Caraglia M, Marra M, Abbruzzese A, Caputi M, Maselli R, Costanzo FS (2005). Mitogen-activated protein kinases and asthma. J Cell Physiol.

[B25] Baraldo S, Faffe DS, Moore PE, Whitehead T, McKenna M, Silverman ES, Panettieri RA, Shore SA (2003). Interleukin-9 influences chemokine release in airway smooth muscle: role of ERK. Am J Physiol Lung Cell Mol Physiol.

[B26] Peng Q, Matsuda T, Hirst SJ (2004). Signaling pathways regulating interleukin-13-stimulated chemokine release from airway smooth muscle. Am J Respir Crit Care Med.

[B27] Cui CH, Adachi T, Oyamada H, Kamada Y, Kuwasaki T, Yamada Y, Saito N, Kayaba H, Chihara J (2002). The role of mitogen-activated protein kinases in eotaxin-induced cytokine production from bronchial epithelial cells. Am J Respir Cell Mol Biol.

[B28] Oltmanns U, Issa R, Sukkar MB, John M, Chung KF (2003). Role of c-jun N-terminal kinase in the induced release of GM-CSF, RANTES and IL-8 from human airway smooth muscle cells. Br J Pharmacol.

[B29] Schnackenberg BJ, Jones SM, Pate C, Shank B, Sessions L, Pittman LM, Cornett LE, Kurten RC (2006). The beta-agonist isoproterenol attenuates EGF-stimulated wound closure in human airway epithelial cells. Am J Physiol Lung Cell Mol Physiol.

[B30] Duan W, Chan JH, Wong CH, Leung BP, Wong WS (2004). Anti-inflammatory effects of mitogen-activated protein kinase kinase inhibitor U0126 in an asthma mouse model. J Immunol.

[B31] Chue SC, Seow CJ, Duan W, Yeo KS, Koh AH, Wong WS (2004). Inhibitor of p42/44 mitogen-activated protein kinase, but not p38 MAPK, attenuated antigen challenge of guinea pig airways in vitro. Int Immunopharmacol.

[B32] Eynott PR, Xu L, Bennett BL, Noble A, Leung SY, Nath P, Groneberg DA, Adcock IM, Chung KF (0125). Effect of an inhibitor of Jun N-terminal protein kinase, SP60 in single allergen challenge in sensitized rats. Immunology.

[B33] Mackenna DA, Dolfi F, Vuori K, Ruoslahti E (1998). Extracellular signal-regulated kinase and c-Jun NH2-terminal kinase activation by mechanical stretch is integrin-dependent and matrix-specific in rat cardiac fibroblasts. J Clin Invest.

[B34] Katsumi A, Naoe T, Matsushita T, Kaibuchi K, Schwartz MA (2005). Integrin activation and matrix binding mediate cellular responses to mechanical stretch. J Biol Chem.

[B35] Li C, Xu Q (2000). Mechanical stress-initiated signal transductions in vascular smooth muscle cells. Cell Signal.

[B36] Iozzo RV (1998). Matrix proteoglycans: from molecular design to cellular function. Annu Rev Biochem.

[B37] Al Jamal R, Roughley PJ, Ludwig MS (2001). Effect of glycosaminoglycan degradation on lung tissue viscoelasticity. Am J Physiol Lung Cell Mol Physiol.

[B38] Roberts CR (1995). Is asthma a fibrotic disease?. Chest.

[B39] de Medeiros MM, da Silva LF, dos Santos MA, Fernezlian S, Schrumpf JA, Roughley P, Hiemstra PS, Saldiva PH, Mauad T, Dolhnikoff M (2005). Airway proteoglycans are differentially altered in fatal asthma. J Pathol.

[B40] Huang J, Olivenstein R, Taha R, Hamid Q, Ludwig M (1999). Enhanced proteoglycan deposition in the airway wall of atopic asthmatics. Am J Respir Crit Care Med.

[B41] Westergren-Thorsson G, Chakir J, Lafreniere-Allard MJ, Boulet LP, Tremblay GM (2002). Correlation between airway responsiveness and proteoglycan production by bronchial fibroblasts from normal and asthmatic subjects. Int J Biochem Cell Biol.

[B42] Liu M, Post M (2000). Invited review: mechanochemical signal transduction in the fetal lung. J Appl Physiol.

[B43] Liu M, Tanswell AK, Post M (1999). Mechanical force-induced signal transduction in lung cells. Am J Physiol.

